# Supplementary material for: An error correction strategy for image reconstruction by DNA sequencing microscopy
Source: Nat Comput Sci. 2024 Jan 22;4(2):119–27. doi: 10.1038/s43588-023-00589-x (PMC10899105; doi:10.1038/s43588-023-00589-x)
Supplement: Supplementary file 1 — Reporting Summary [file 43588_2023_589_MOESM1_ESM.pdf]

nature portfolio

Corresponding author(s): Björn Högberg

Last updated by author(s): Dec 8, 2023

Reporting Summary

Nature Portfolio wishes to improve the reproducibility of the work that we publish. This form provides structure for consistency and transparency in reporting. For further information on Nature Portfolio policies, see our Editorial Policies and the Editorial Policy Checklist.

Statistics

For all statistical analyses, confirm that the following items are present in the figure legend, table legend, main text, or Methods section.

n/a	Confirmed	
☐	☒	The exact sample size (*n*) for each experimental group/condition, given as a discrete number and unit of measurement
☒	☐	A statement on whether measurements were taken from distinct samples or whether the same sample was measured repeatedly
☒	☐	The statistical test(s) used AND whether they are one- or two-sided<br>*Only common tests should be described solely by name; describe more complex techniques in the Methods section.*
☒	☐	A description of all covariates tested
☒	☐	A description of any assumptions or corrections, such as tests of normality and adjustment for multiple comparisons
☐	☒	A full description of the statistical parameters including central tendency (e.g. means) or other basic estimates (e.g. regression coefficient) AND variation (e.g. standard deviation) or associated estimates of uncertainty (e.g. confidence intervals)
☒	☐	For null hypothesis testing, the test statistic (e.g. *F*, *t*, *r*) with confidence intervals, effect sizes, degrees of freedom and *P* value noted<br>*Give P values as exact values whenever suitable.*
☒	☐	For Bayesian analysis, information on the choice of priors and Markov chain Monte Carlo settings
☒	☐	For hierarchical and complex designs, identification of the appropriate level for tests and full reporting of outcomes
☒	☐	Estimates of effect sizes (e.g. Cohen's *d*, Pearson's *r*), indicating how they were calculated

Our web collection on statistics for biologists contains articles on many of the points above.

Software and code

Policy information about availability of computer code

Data collection	Data was generated using only custom code provided with the manuscript, and is made available at 10.5281/zenodo.10256692. Simulation code was ran in python v3.9.12 using numba v.0.53.1 and numpy v1.22.4.
Data analysis	Data was analysed using custom code provided with the manuscript, availble from 10.5281/zenodo.10256692 and also from https://github.com/Alexamk/minipath/). Analysis code was ran in python v3.9.12 using numba v.0.53.1, numpy v1.22.4, pandas v.1.4.4, scikit-learn v1.1.3, scipy v1.9.3, and seaborn v.0.11.2.<br><br>Part of the code used to create the microscopy reconstructions is slightly adapted from previously published code, available from https://github.com/jaweinst/dnamic.

For manuscripts utilizing custom algorithms or software that are central to the research but not yet described in published literature, software must be made available to editors and reviewers. We strongly encourage code deposition in a community repository (e.g. GitHub). See the Nature Portfolio guidelines for submitting code & software for further information.

Data

Policy information about availability of data

All manuscripts must include a data availability statement. This statement should provide the following information, where applicable:

- Accession codes, unique identifiers, or web links for publicly available datasets
- A description of any restrictions on data availability
- For clinical datasets or third party data, please ensure that the statement adheres to our policy

The input for the simulations and the data generated by the simulations are available in the Zenodo repository: 10.5281/zenodo.10256692. The previously published raw experimental data is available at the Sequencing Read Archive (project number PRJNA487001, sample 3). Source Data for Figures 2, 4, and 5, and for Extended Data Figure 1-10 is available with this manuscript.

Human research participants

Policy information about studies involving human research participants and Sex and Gender in Research.

Reporting on sex and gender	n/a
Population characteristics	n/a
Recruitment	n/a
Ethics oversight	n/a

Note that full information on the approval of the study protocol must also be provided in the manuscript.

Field-specific reporting

Please select the one below that is the best fit for your research. If you are not sure, read the appropriate sections before making your selection.

☒ Life sciences ☐ Behavioural & social sciences ☐ Ecological, evolutionary & environmental sciences

For a reference copy of the document with all sections, see nature.com/documents/nr-reporting-summary-flat.pdf

Life sciences study design

All studies must disclose on these points even when the disclosure is negative.

Sample size	Simulations were run once per condition, since we expected variation when changing simulation parameters not to affect the overall results. We instead fixed one simulation parameter at a time and varied the others to obtain multiple samples per parameter, from which means were calculated. No experimental data was collected for which choosing a sample size may have been appropriate.
Data exclusions	Reconstructions were left out of the data analysis if the largest connected component of the corresponding neighborhood graph contained less than 80% of all nodes, since the reconstruction accuracy could not be accurately determined for these reconstructions
Replication	Simulations were not repeated, since we expected variation when changing simulation parameters not to affect the overall results. We instead fixed one simulation parameter at a time and varied the others to obtain multiple samples per parameter. No experimental data was collected for which replication may have been appropriate.
Randomization	No experiment was performed for which randomization was applicable.
Blinding	No experiment was performed for which blinding was applicable.

Reporting for specific materials, systems and methods

We require information from authors about some types of materials, experimental systems and methods used in many studies. Here, indicate whether each material, system or method listed is relevant to your study. If you are not sure if a list item applies to your research, read the appropriate section before selecting a response.

Materials & experimental systems

n/a	Involved in the study	
☒	☐	Antibodies
☒	☐	Eukaryotic cell lines
☒	☐	Palaeontology and archaeology
☒	☐	Animals and other organisms
☒	☐	Clinical data
☒	☐	Dual use research of concern

Methods

n/a	Involved in the study	
☒	☐	ChIP-seq
☒	☐	Flow cytometry
☒	☐	MRI-based neuroimaging
