# Supplementary figures and images for: An error correction strategy for image reconstruction by DNA sequencing microscopy
Source: Nat Comput Sci. 2024 Jan 22;4(2):119–27. doi: 10.1038/s43588-023-00589-x (PMC10899105; doi:10.1038/s43588-023-00589-x)

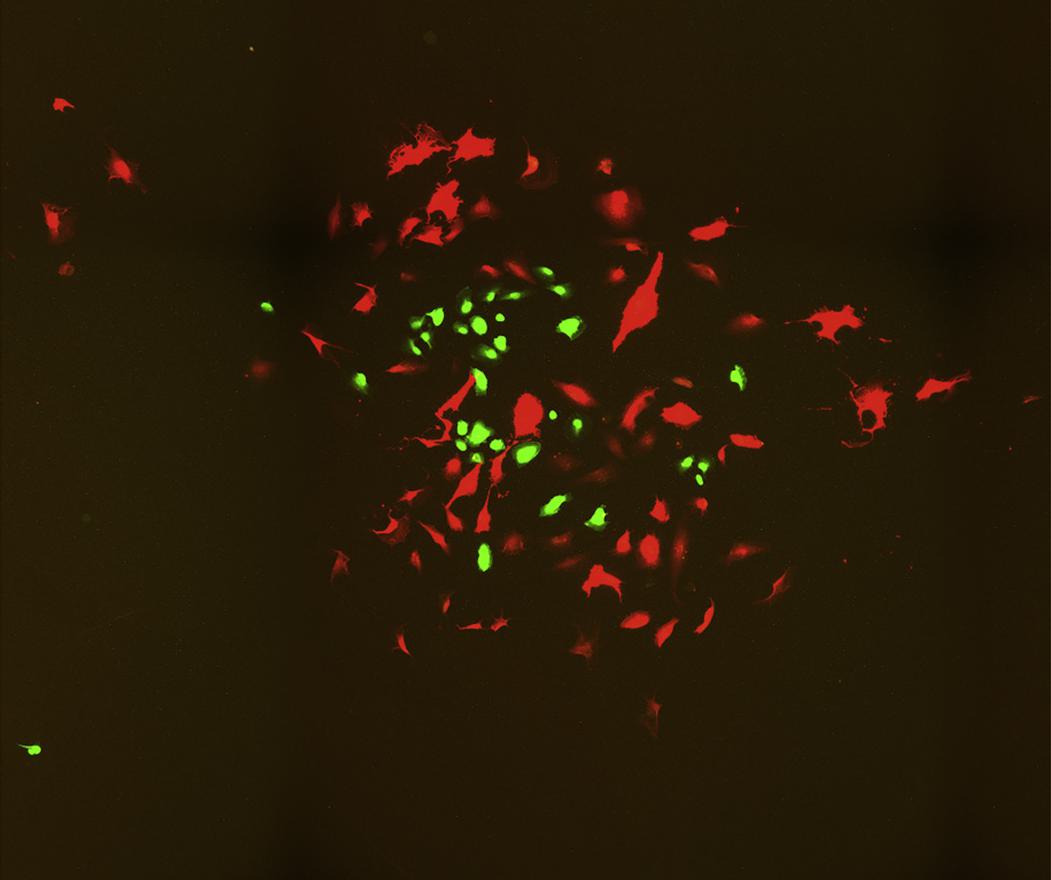

Supplement: Supplementary file 5 — Raw images before overlay and numerical source data. [file 43588_2023_589_MOESM5_ESM.zip › Fig5_source_data/Fig5A.jpg]

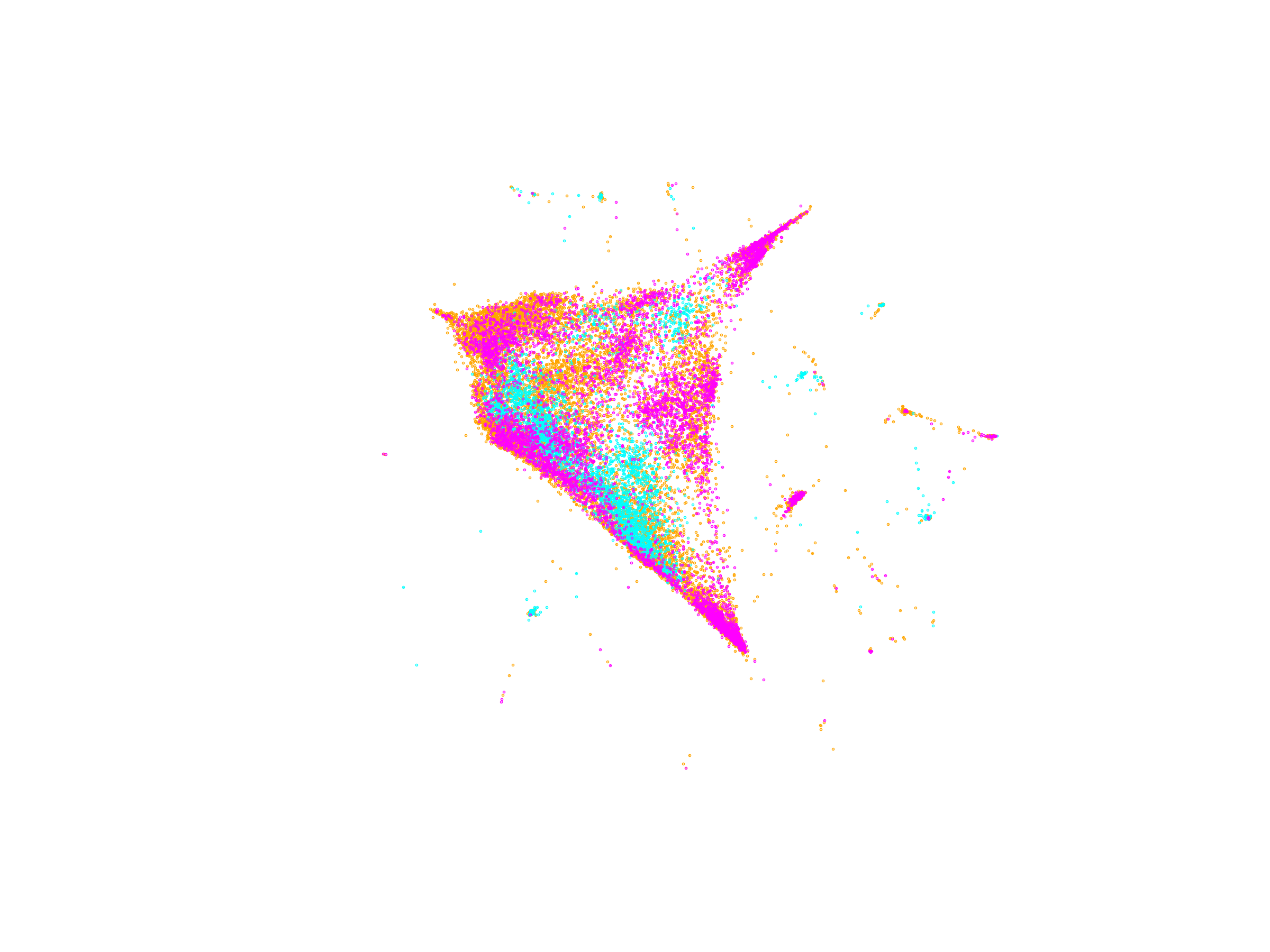

Supplement: Supplementary file 5 — Raw images before overlay and numerical source data. [file 43588_2023_589_MOESM5_ESM.zip › Fig5_source_data/Fig5B_reconstruction.png]

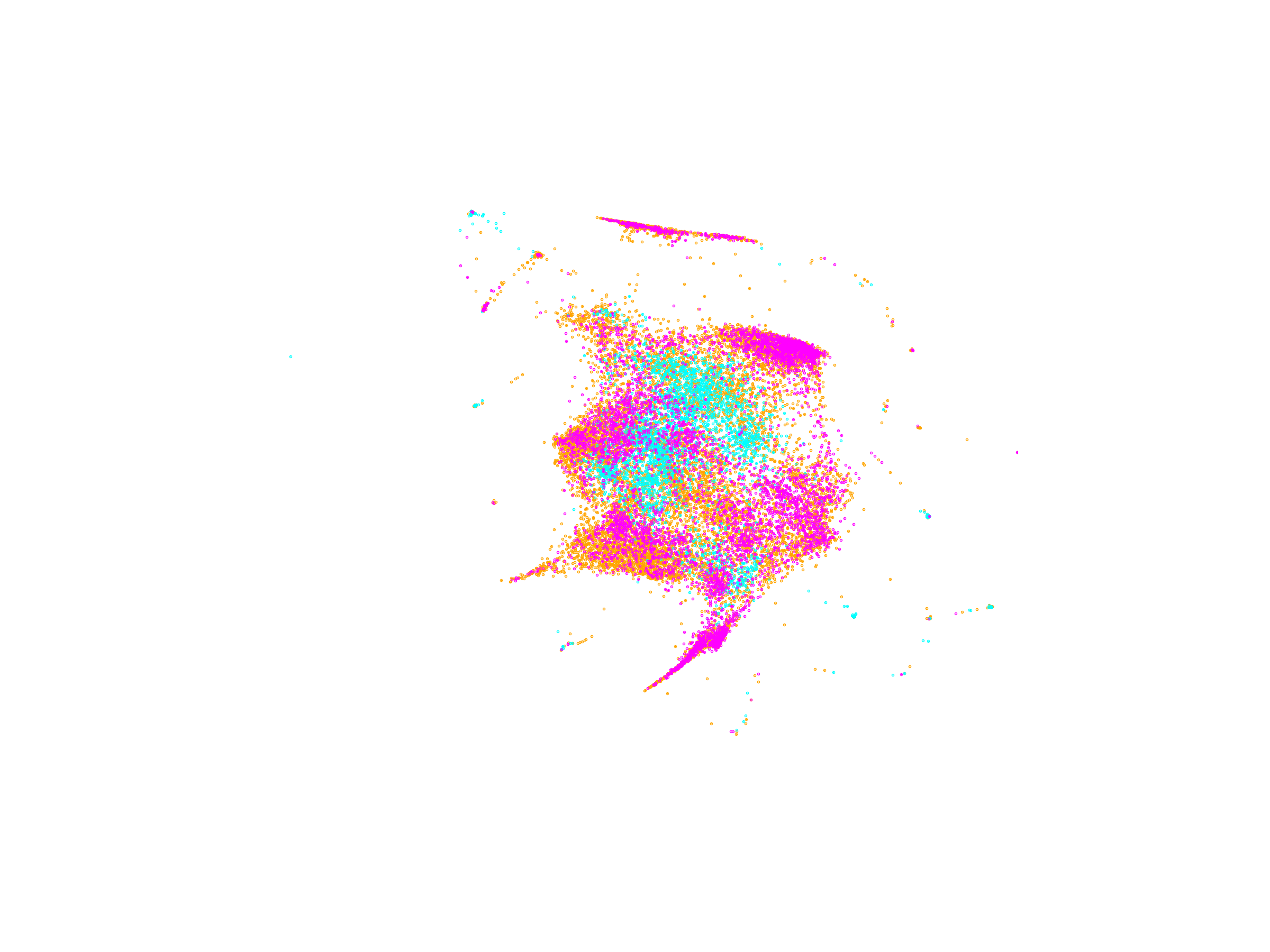

Supplement: Supplementary file 5 — Raw images before overlay and numerical source data. [file 43588_2023_589_MOESM5_ESM.zip › Fig5_source_data/Fig5C_reconstruction.png]

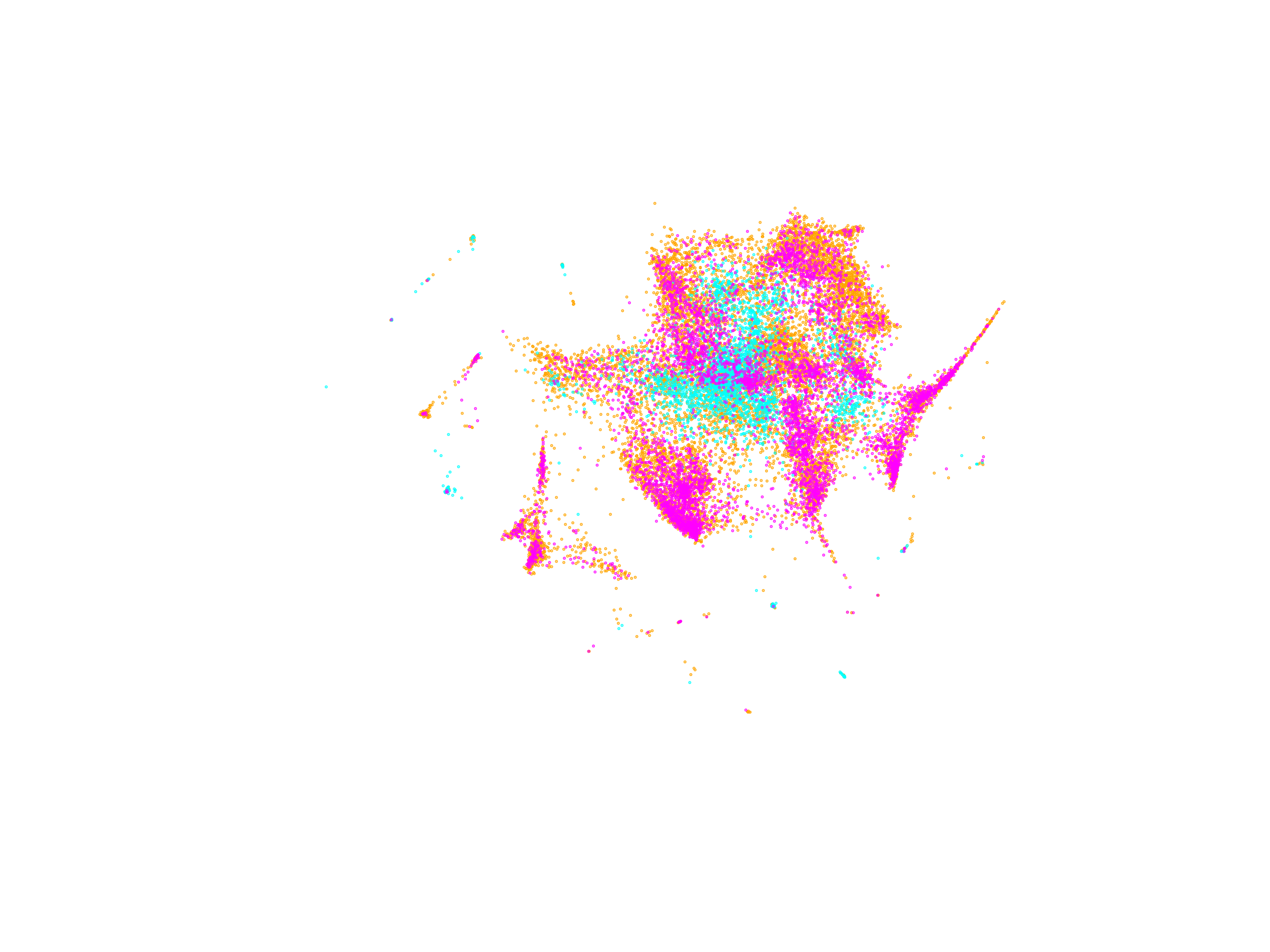

Supplement: Supplementary file 5 — Raw images before overlay and numerical source data. [file 43588_2023_589_MOESM5_ESM.zip › Fig5_source_data/Fig5D_reconstruction.png]

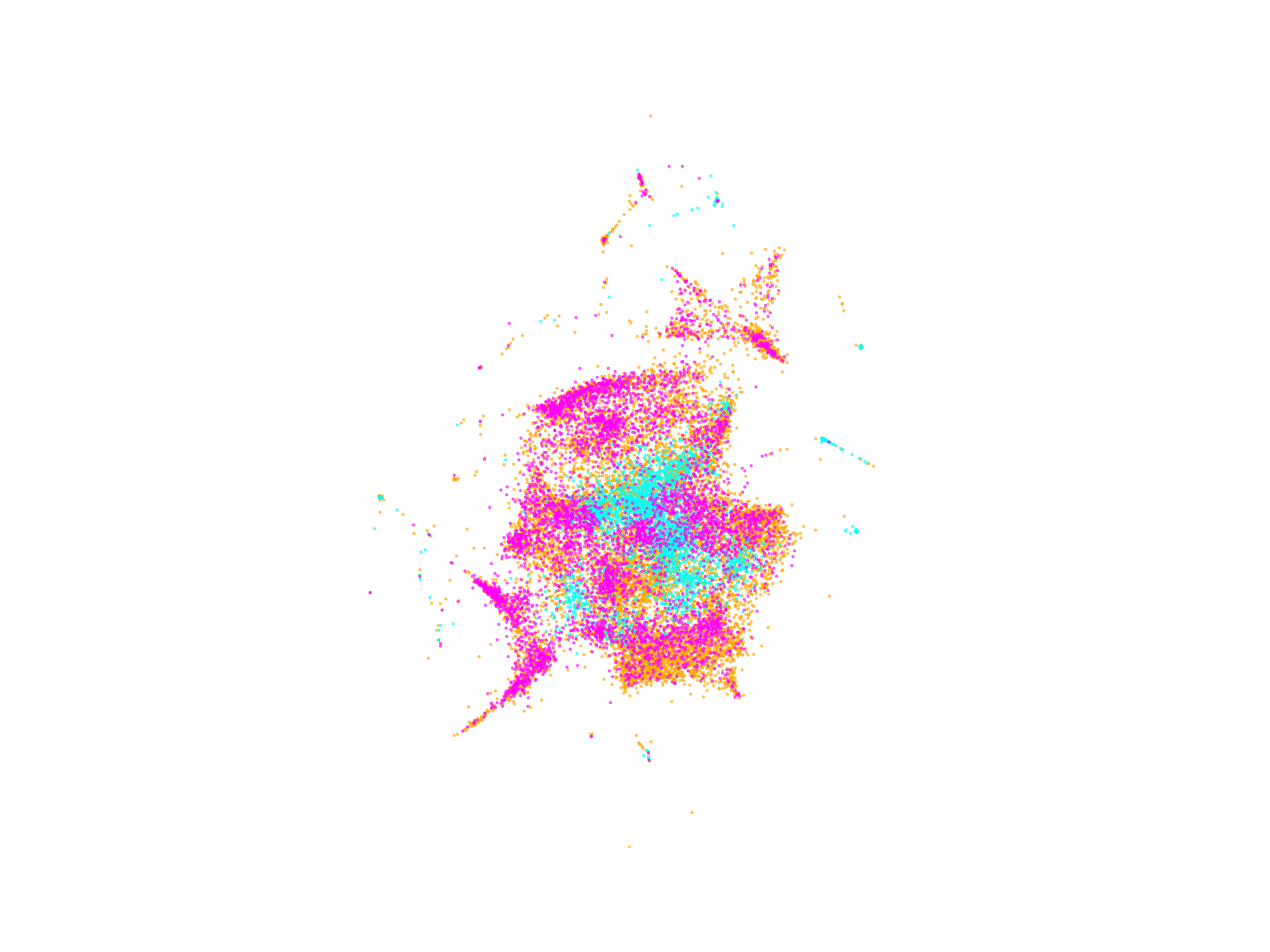

Supplement: Supplementary file 5 — Raw images before overlay and numerical source data. [file 43588_2023_589_MOESM5_ESM.zip › Fig5_source_data/Fig5E_reconstruction.png]

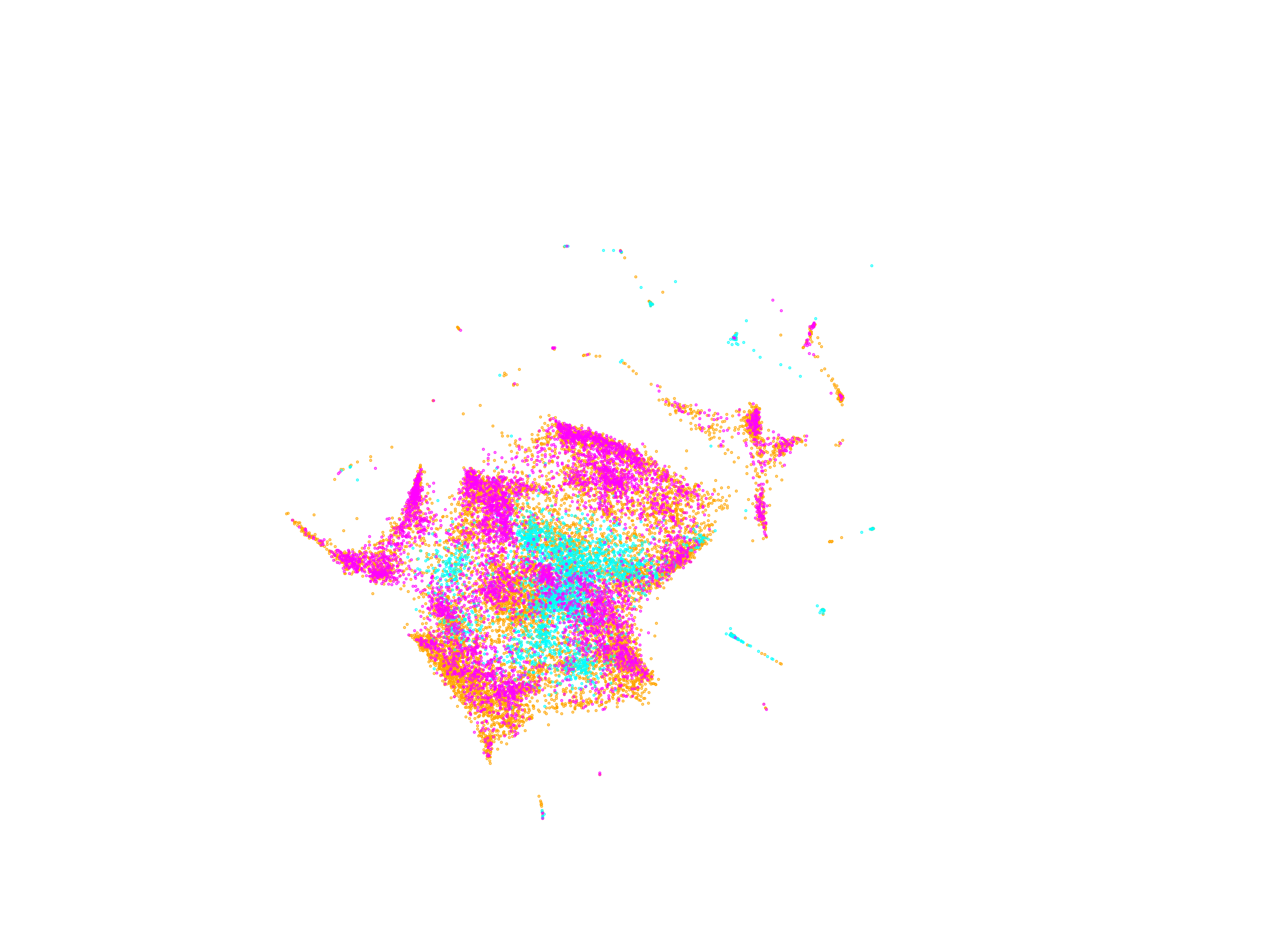

Supplement: Supplementary file 5 — Raw images before overlay and numerical source data. [file 43588_2023_589_MOESM5_ESM.zip › Fig5_source_data/Fig5F_reconstruction.png]
